# A rare case of glomangiopericytoma in the nasal cavity: A case report in light of recent literature

**DOI:** 10.1016/j.amsu.2022.103685

**Published:** 2022-04-28

**Authors:** Firas K. Almarri, Abdullah M. Alnatheer, Muath K. Abuhaimed, Abeer A. Albathi, Abdulmalik Q. Alqahtani, Tariq Tatwani

**Affiliations:** a College of Medicine, Imam Mohammed Ibn Saud Islamic University, Riyadh, Saudi Arabia; b Department of Otorhinolaryngology – Head and Neck Surgery, Prince Sultan Military Medical City, Riyadh, Saudi Arabia; c Department of Pathology , Prince Sultan Military Medical City, Riyadh, Saudi Arabia

**Keywords:** Glomangiopericytoma, Rare, Nasal cavity, Sinonasal tumor, Histology, Case report

## Abstract

**Introduction and importance:**

Glomangiopericytoma (GPC) is a rare sinonasal tumor that behaves benignly with a long overall survival rate. It accounts for fewer than 0.5% of all sinonasal tumors.

**Case presentation:**

We report the case of a 64-year-old man who presented with recurrent episodes of epistaxis. Rhinoscopy revealed a left posterior nasal septal mass with active oozing. Computed tomography (CT) showed a well-defined soft tissue lesion in the left nasal cavity measuring 1.95 × 1.51 cm. Complete endoscopic resection was successfully performed. Histopathological findings favored the diagnosis of GPC as it revealed tumor cells positive for smooth muscle actin and β-catenin with immunopositivity for CD34.

**Clinical discussion:**

Presenting symptoms of GPC are predominated by epistaxis and nasal obstruction. Since CT and MRI merely lead to a presumptive diagnosis, histopathological findings are indispensable. Complete surgical excision of GPC remains the treatment of choice with excellent prognosis, especially when immunohistochemistry is positive for actin and CD34 immunostaining is negative.

**Conclusion:**

GPC is a rare indolent tumor of pericytes that has a macroscopic appearance of a nasal polyp, which may result in uncertainty in the initial diagnosis. In most cases, GPC warrants only local excision. This case report adds to the literature and helps galvanize the developing clinical guidelines for diagnosis and treatment.

## Highlights

• Glomangiopericytoma (GPC) is a rare sinonasal tumor that behaves benignly with a long overall survival rate.
• GPC has a macroscopic appearance of a nasal polyp, resulting in uncertainty in the initial diagnosis.
• The utilization of histopathological findings to diagnose GPC is indispensable.
• Complete surgical excision is the mainstay of treatment.
• Regular postoperative lifelong follow-up is strongly recommended.

## 1 Introduction

Glomangiopericytoma (GPC) is a rare sinonasal tumor emerging from Zimmerman's pericytes that surround the capillaries and accounts for fewer than 0.5% of all sinonasal neoplasms [1,2]. Notably, GPC was previously reported as hemangiopericytoma and was initially described in 1942 by Stout and Murray as a soft tissue tumor with distinctive vascular proliferation, including branching vessels and small vessel perivascular hyalinization [3]. In 2005, GPC was regarded by the World Health Organization (WHO) as a borderline and low malignant potential soft tissue tumor of the nose and paranasal sinuses with an excellent overall survival rate [4,5]. The etiology of GPC remains unknown. Hypertension, trauma, pregnancy, and long-term use of corticosteroids are considered possible causes [2]. We report a case of GPC arising in the left sinonasal cavity manifesting as recurrent episodes of epistaxis treated by endoscopic excision. Since this is an uncommon disease that has been rarely reported, this report contributes to the current literature. Furthermore, it will help galvanize future guidelines for both treatment and management. This case report has been reported in line with the SCARE 2020 criteria [6].

## 2 Case presentation

**Fig. 1:**
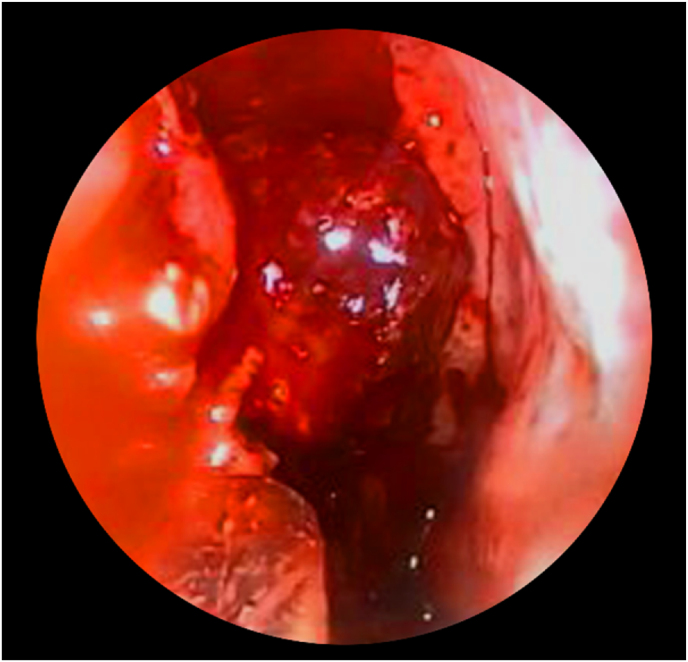
Endoscopic view of the tumor. The lesion is displacing the nasal septum medially and the left middle turbinate superolaterally.

**Fig. 2:**
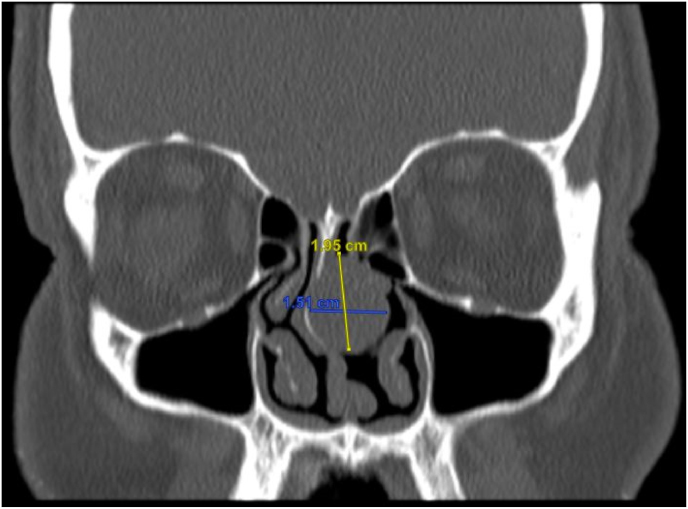
Computed tomography scan (coronal view) of the paranasal sinuses showing a well-defined soft tissue density lesion in the left nasal cavity, measuring 1.95 × 1.51 cm.

**Fig. 3:**
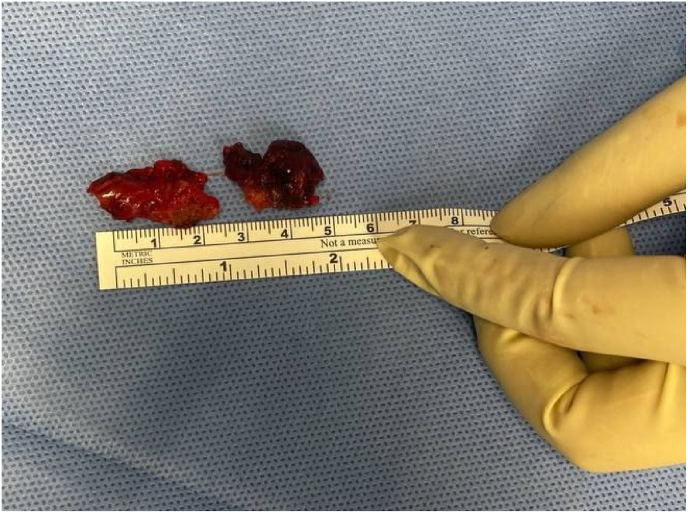
Surgical specimen after excision.

**Fig. 4:**
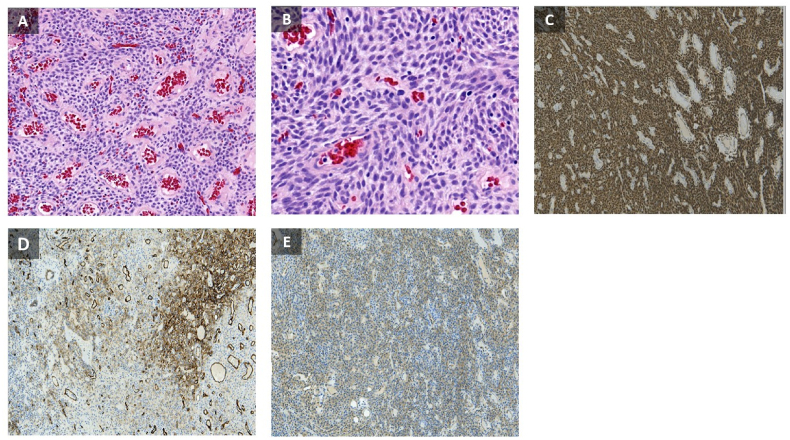
Low power (**A**) and high power (**B**) magnification depicting a storiform/fascicular growth of spindle to oval monotonous tumor cells with perivascular hyalinization compatible with glomangiopericytoma. (**C**) Strong diffuse nuclear expression of β-catenin. (**D**) Immunohistochemical staining for tumor cells showing CD34 to be focally positive. (**E**) The tissue if focally, weakly positive for smooth muscle actin; supporting myoid differentiation of glomangiopericytoma.

**Fig. 5:**
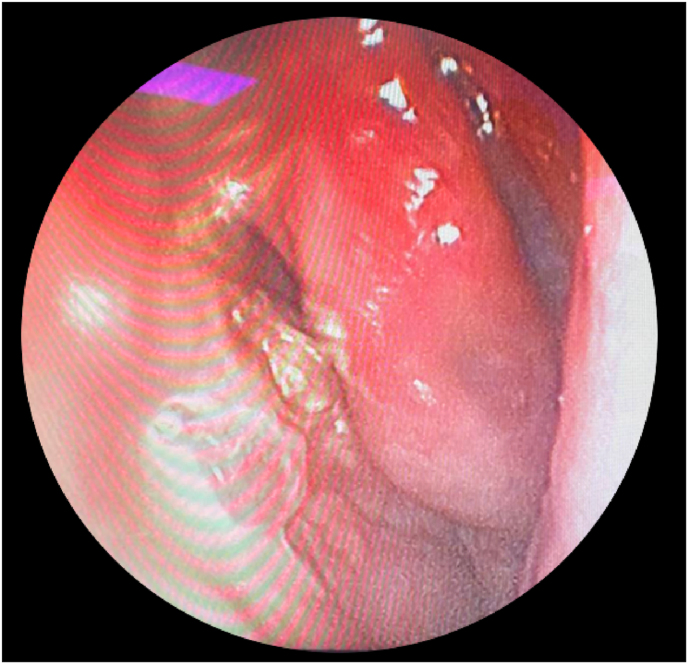
Endoscopic follow-up image after two months showing healed mucosa in the nasal cavity and minimal incrustation over the septum.

A 64-year-old man presented to our department with a history of diabetes mellitus, hypertension, and septoplasty conducted 6 years previously. He had presented with epistaxis to the primary health care emergency center multiple times over 3 years. Multiple anterior nasal packing at different times helped control the epistaxis initially. Thereafter, the patient presented to our emergency department in a tertiary facility with active epistaxis. Transnasal flexible scope showed left posterior nasal septal mass with active oozing from the mass, which was managed by a merocele nasal pack and surgicel absorbable hemostat to stop the bleeding (Fig. 1). Computed tomography (CT) of the paranasal sinuses without contrast reported a well-defined soft tissue density lesion without any calcification in the left nasal cavity, which was adherent to the septum causing medial displacement of the nasal septum, superior lateral displacement of the left middle nasal turbinate, and lateral deviation of the left uncinate process narrowing the left ostiomeatal complex. The lesion measured 1.95 × 1.51 cm in maximum. Other associated CT findings included a mild mucosal thickening noted in the nasal cavity along the nasal septum. The nasal septum appeared deviated towards the right with bony spur formation. Minimal mucosal thickening was noted in the bilateral maxillary sinuses. Soft tissue opacification of the left frontal sinus and frontoethmoidal recess were noted. Minimal mucosal thickening was noted in the bilateral ethmoid air cells (Fig. 2). Based on the recorded history, physical examination, and CT findings, the patient was considered for endoscopic septal mass excision under general anesthesia planned to be performed by a specialist and a senior resident under the supervision of a consultant. For surgical excision, a Killian incision was performed anterior to the mass. Upon dissection of the flap, adhesions due to the previous surgery were noticed. Using a Colorado tip, a wide local excision was performed, and the specimen was sent for tissue sampling (Fig. 3). Intraoperatively, the septum was not involved by the mass; thereby, refuting the initial CT scan assessment. The patient was sent to recovery postoperatively without any packing. Histopathologic examination revealed spindle cell lesions that are most likely compatible with a sinonasal GPC. Immunohistochemistry reported tumor cells that are positive for smooth muscle actin and β-catenin (nuclear staining) and focally positive for CD34 (Fig. 4). The patient was seen 2 weeks postoperatively and then 6 weeks later, with no bleeding history during that period. Upon reexamination, incrustations were noted all over the surgical site and debrided (Fig. 5). The patient is under regular close follow-up with no signs of recurrence to date.

## 3 Discussion

GPC of the nasal cavity constitutes a rarity among all nasal neoplasms as it accounts for less than 0.5% of nasal tumors [7]. GPC seems to occur in the late adult life (60s or 70s) with a slight preponderance in women and unknown pathogenesis [8]. Nevertheless, it has been alleged that its etiology may be due to high vascularization caused by either previous trauma, hypertension, pregnancy, and long-term use of corticosteroids [2]. In our case, the patient was in his 60s, hypertensive, and interestingly, underwent septoplasty 6 years previously; yet the etiology of the patient's tumor is unknown. Moreover, there are few reported cases and only one in this region [9]. This could be attributed to the GPC being missed or underdiagnosed, since the tumors are indolent and harbor features similar to those found in sinonasal polyps, for which it might be mistaken [10]. This observation indicates a need to emphasize the importance of timely and accurate referral to otorhinolaryngology specialists for cases presenting with recurrent epistaxis, unresolved nasal obstruction, and headache, which are the most common presenting symptoms of GPC [11]. Diagnosing GPC can be laborious, often requiring endoscopy, CT, and MRI to assess the size, extent, and characteristics of the tumor, allowing adequate preoperative care [12]. Plausibly, GPC's radiographic findings may be ambiguous given the resemblance to nasal polyps on a CT scan (Fig. 2). On imaging, GPC presents as an enhancing soft tissue mass that is spherical or lobulated in shape and bony erosive in nature [13]. On MRI, it shows a varied T2 appearance, including T2 hyperintensity and intermediate signal [13]. Although these investigations appear exhaustive, they permit only a presumptive diagnosis; thus, histopathological tissue diagnosis remains the gold standard for definitive diagnosis [14]. In general, a GPC portrays a uniform epithelioid cell histologically with prominent perivascular arrangements and stains positively for actin with conflicting CD34 immunostaining [15]. Regarding the treatment options, complete local excision remains the preferred management option with an excellent 5-year survival rate of 88% [16]. The recurrence rate is reportedly 17% [17]. This percentage is thought to increase by inadequate surgical excision that can be subsequently managed by additional surgery [16,18]. Therefore, regular postoperative lifelong follow-up is strongly recommended. In our present case, the tumor was ensured to be wholly resected endoscopically to prevent any local recurrence possibilities.

In addition to previous reviews, we conducted a literature review of cases published between 2018 and 2022 to investigate the characteristics, findings of CT and MRI scans, surgical approaches, and the treatment outcomes of patients with GPC [11,19]. Of the total 12 cases reported within the timeframe, the majority were men (n = 8/12), with a mean age of 59 years. The presenting symptoms in these cases were predominated by epistaxis and nasal obstruction, consistent with our case. Almost all cases required endoscopic sinus surgery for the excision of the tumor with no apparent postoperative complications in almost all cases (Table 1). CT and MRI imaging were used to locate and evaluate the nature of the mass and its disposed effect on the surrounding structures. Preoperatively, CT was the mainstay imaging modality in all cases, while MRI was utilized in three only cases, both with diverse findings (Table 2). This again strongly indicates the necessity of tissue histopathology for final diagnosis. In terms of prognosis, a systematic review revealed that actin and CD34 might be employed as independent prognostic markers in GPCs, as immunopositivity for actin and immunonegativity for CD34 contribute to an overall longer survival rate [15].

**Table 1:** Review of all reported cases of GPC in the English literature between 2018 and 2022.

Case No.	Author	Year of publication	Age	Gender	Location	Presenting symptoms	Surgical technique	Surgery complications	Recurrence	Mean follow-up (months)
1	Takashi Anzai et al. [20]	2018	68	M	Nasal cavity	Epistaxis	ESS	Not mentioned	Not mentioned	N/A
2	Chan‐Jung Changa et al. [19]	2018	21	M	Maxillary sinus, middle meatus, ethmoid sinus, frontal recess, and frontal sinus.	Epistaxis, nasal obstruction, anosmia. diplopia and proptosis,	Navigation-assisted endoscopic	None	No	12
3	Sana Sheikh et al. [21]	2018	23	M	Nasal cavity	Nasal obstruction and epistaxis	Endonasal endoscopic laser-assisted resection	None	No	18
4	Michihisa Kono et al. [11]	2018	74	F	Nasal cavity	Nasal obstruction and epistaxis	ESS	Not mentioned	Not mentioned	N/A
5	Nitin Sharmaa et al. [22]	2019	65	M	Sphenoethmoidal recess	Epistaxis, pain	ESS	Not mentioned	No	9
6	Yutaro Saito et al. [18]	2019	71	M	Nasal cavity	Nasal obstruction	ESS	None	No	13
7	Larry Shemen et al. (case 1) [23]	2020	62	F	superior turbinate	Headache	ESS	Not mentioned	No	12
8	Larry Shemen et al. (case 2) [23]	2020	79	M	Middle meatus.	Nasal obstruction and epistaxis	endoscopic sinus surgery with the assistance of an image guidance system	Not mentioned	No	12
9	Shayan Khalid Ghaloo et al. [24]	2020	70	M	Nasal cavity	Nasal obstruction and epistaxis	ESS	None	No	3
10	A. Chaouki et al. [25]	2021	47	F	Anterior naris	Nasal obstruction and epistaxis	ESS	Bleeding	No	24
11	Al-Jobory et al. [12]	2021	60	M	Left ethmoid sinus	None (incidental finding)	ESS	None	NO	21
12	Christopher S. Hong et al. [26]	2022	69	F	Nasal cavity	Epistaxis	ESS	None	Not mentioned	N/A

M, male; F, female; ESS, endoscopic sinus surgery.

**Table 2:** Radiological findings of reported cases of GPC.

Author	Modality	Findings
Takashi Anzai et al. [20]	CT	Small mass (about 5 mm) in the right nasal cavity that had arisen from the septal wall.
Chan‐Jung Changa et al. [19]	CT	A mass lesion occupying the left maxillary sinus, middle meatus, ethmoid sinus, frontalrecess, and frontal sinus. Obvious mass effect with the surrounding structure deviation was observed, and bone destruction was highly suspected.
Sana Sheikh et al. [21]	CT/MRI	CT: A well-defined soft-tissue density area measuring 2.1 × 1.9 × 1.5cm arising from nasal septum extending into right nasal cavity laterally abutting the right middle turbinate and extending inferiorly up to right inferior turbinate.
		MRI: A homogenous enhancement in the lesion with absence of nodal involvement.
Michihisa Kono et al. [11]	CT	A mass occupying the right nasal cavity with strong enhancement.
Nitin Sharmaa et al. [22]	CT	Polypoidal mucosal thickening in sphenoid sinus with complete opacification. There was hyperdensity without any abnormal enhancement, but mild focal extension of soft tissue was seen in sphenoethmoidal recess left side and protruding into nasopharynx.
Yutaro Saito et al. [18]	CT	Showed a low-density, homogeneous lesion occupying the left nasal cavity.
Larry Shemen et al. (case 1) [23]	CT	Showed a 2.7cm mass obstructing the sphenoid sinus.
Larry Shemen et al. (case 2) [23]	CT	Left sphenoethmoid opacification with polypoid degeneration of the other sinuses.
Shayan Khalid Ghaloo et al. [24]	CT	An enhancing lesion in the right nasal cavity posteriorly, measuring 16 × 10 mm. The lesion lay within the anterior ethmoid air cells, adherent medially to the nasal septum and laterally to the right lateral wall of the nasal cavity. (Fig. 1). It was seen obstructing the passage of right frontal and ethmoid sinuses.
A. Chaouki et al. [25]	CT	A lesion involving the left nasal cavity, with a soft tissue density (70 UH) measuring 50 × 16 mm, widely infiltrative (left nasal turbinates, uncinate process, left half of nasopharynx and palatine bone), with a posterior left ethmoidal sinus thickening.
Al-Jobory et al. [12]	CT/MRI	CT: Demonstrated a mass isodense to soft tissue, without any calcification. The 2.5 cm left ethmoid mass demonstrated arterial enhancement, eroding the ethmoid cribriform plate and left lateral lamella (Fig. 1A and B). However, there was no adjacent periosteal reaction or hyperostosis.
		MRI: the mass was isointense to brain parenchyma on T1 and T2 sequences with no restricted diffusion, and homogenous contrast enhancement. There was no dural enhancement or thickening. There was only minimal mass effect, and no adjacent cerebral edema.
Christopher S. Hong et al. [26]	CT/MRI	CT: A hypodense, partially cystic mass in the right nasal cavity causing obstruction and opacification of the right posterior ethmoid air cells and sphenoid sinus, as well as bony remodeling of the ipsilateral cribriform plate and lateral lamella.
		MRI: Demonstrated a 3.3 × 1.2-cm enhancing soft tissue mass, protruding into the ipsilateral sphenoid sinus without definite intracranial extension.

CT, computed tomography; MRI, magnetic resonance imaging.

## 4 Conclusion

GPC is a rare indolent sinonasal tumor with low malignant potential. The diagnosis of GPC necessitates a high index of suspension because of its rarity and ambiguity of clinical and radiological signs. Furthermore, our case report supports the medical evaluation of GPC. Histopathological tissue sampling is the diagnosis of certitude. The mainstay of treatment is complete surgical excision with regular monitoring. Consistent reporting of rare cases of GPC is crucial as it provides guidance for diagnosis, follow-up, and treatment outcome, since GPC is rarely discussed in the medical literature.

## Ethical approval

The ethical committee approval was not required given the article type is a case report. However, the written consent to publish the clinical data of the patients were given and is available to check by the handling editor if needed.

## Sources of funding

This case report did not receive any specific grant from funding agencies in the public, commercial, or not-for-profit sectors.

## Consent

Written informed consent was obtained from the patient for publication of this case report and accompanying images. A copy of the written consent is available for review by the Editor-in-Chief of this journal on request.

## Author contribution

AMA, MKA, and FKA conceived the study design. AMA and FKA performed the literature review. AMA, MKA, AAA, and TT prepared the components of the case presentation. FKA drafted the manuscript. AA and FKA designed the tables. MKA, AAA, and TT performed surgery and provided bedside care. AQA performed histopathological diagnosis. All authors approved the final version of the manuscript.

## Registration of research studies

This is a case report.

## Guarantor

Firas K. Almarri, College of Medicine, Imam Mohammed Ibn Saud Islamic University, Riyadh, Saudi Arabia ORCID: https://orcid.org/0000-0002-8981-411X Tel: +966548104118 Email: FirasAlmarri@gmail.com.

### Patient perspective

Our patient responded positively to the surgical and medical management offered by the medical team as his condition improved drastically. Therefore, he is grateful to the Otorhinolaryngology-Head & Neck Surgery department for assisting him in his recovery.

## Provenance and peer review

Not commissioned, externally peer-reviewed.

## Declaration of competing interest

The authors state that they have no conflicts of interest for this case report.
